# Arginine inhibits cross-kingdom interactions and synergistic cariogenicity between *Streptococcus mutans* and *Candida albicans*

**DOI:** 10.3389/fcimb.2025.1633342

**Published:** 2025-10-14

**Authors:** Hong-yu Gao, Hao Yang, Hong-mei Wang, Hao-ming Li, Yan-song Ma, Yu-xing Bai

**Affiliations:** Department of Orthodontics, Beijing Stomatological Hospital, Capital Medical University, Beijing, China

**Keywords:** *Streptococcus mutans*, *Candida albicans*, arginine, cariogenicity, cross-kingdom interaction

## Abstract

**Introduction:**

*Streptococcus mutans* and *Candida albicans* are common pathogenic organisms from the oral microbial community, and are associated with the pathogenesis of caries. We investigated the repressive effects of arginine on the cross-kingdom interactions and synergistic cariogenicity between *S. mutans* and *C. albicans*.

**Methods:**

The effect of arginine on the growth of *S. mutans* and *C. albicans* in the planktonic state was reflected by analyzing growth curves and pH measurements. Biofilm biomass was measured using growth curves, crystal violet staining, and colony-forming unit measurements; fluorescence *in situ* hybridization indicated the physical relationship between *S. mutans* and *C. albicans* in biofilms. The cariogenic properties of dual-species biofilms were analyzed through extracellular polysaccharide and lactic acid production assessments.

**Results:**

Arginine inhibited the planktonic growth and biofilm formation of *S. mutans* and *C. albicans*, with reduced biofilm formation, biomass, and physical adhesion between strains. Moreover, arginine suppressed the production of extracellular polysaccharides and lactic acid. In addition, short-term arginine treatment effectively inhibited the growth of *S. mutans* and *C. albicans*.

**Conclusion:**

L-Arginine inhibited both mono- and dual-species growth of *S. mutans* and *C. albicans*. Thus, L-arginine may serve as a novel approach to inhibit the cross-kingdom interactions and synergistic cariogenicity of *S. mutans* and *C. albicans*.

## 1 Introduction

The most prevalent major oral disease is deciduous tooth caries, which is estimated to affect 43% of the world’s population, followed by dental caries of permanent dentition, with an average global prevalence of 29%, according to the World Health Organization (Cherian et al., 2023). Untreated dental caries affects masticatory function, speech, smile characteristics, and psychology, as well as quality of life. Plaque attached to the tooth surface is the main biological factor leading to dental caries. Numerous cariogenic bacteria, including *Streptococcus mutans*, *Actinomyces gerencseriae*, *Propionibacterium acidifaciens*, and *Scardovia wiggsiae*, have been identified in recent studies, revealing multiple observed synergistic interactions (Jenkinson, 2011; Pang et al., 2021).

In recent years, fungi, particularly *Candida albicans*, have garnered attention for their cariogenic potential. Recent studies have proposed that *C. albicans* is also involved in the formation of caries and that *S. mutans* and *C. albicans* have a synergistic cariogenic effect. *S. mutans* and *C. albicans* co-exist in high enrichment and have been co-detected in a variety of oral disease models, such as early childhood caries (Xiao et al., 2018; Lu et al., 2023), root caries (Du et al., 2021), white spot lesions (Klaus et al., 2016; Beerens et al., 2017; Yang et al., 2023), and denture stomatitis (Baena-Monroy et al., 2005; Clemente et al., 2023). Research has demonstrated that cross-kingdom interactions between cariogenic bacteria and fungi enhance the formation of cariogenic biofilms (Koo et al., 2018; Wan et al., 2021). The synergistic pathogenicity of *S. mutans* and *C. albicans* is manifested in physical adhesion, metabolic promotion, acid production, and cross-feeding (Du et al., 2022; Li et al., 2023). *S. mutans* and *C. albicans* utilize sucrose to synthesize exopolysaccharides and produce massive cross-kingdom biofilms. Moreover, their symbiotic effects promote carbohydrate metabolism and upregulate the expression of virulence genes, increasing the biomass of, enhancing the active metabolism in, decreasing the pH of, and increasing the cariogenicity of the dual-species biofilm of *S. mutans* and *C. albicans* (He et al., 2017; Xiao et al., 2023).

Approaches to inhibit cross-kingdom interactions between *S. mutans* and *C. albicans* may be important in preventing dental caries. In most clinical treatments for infections caused by microorganisms, antibiotics or antifungal drugs are used; however, resistance and dysbiosis caused by broad-spectrum antimicrobial drugs limit their use in the treatment of biofilm-associated infections. The development of biofilm-targeted therapeutic strategies to disrupt the growth of polymicrobial biofilms is urgently needed to combat oral diseases and maintain oral health; many approaches, such as natural compounds, chemical compounds, nanomaterials, and photodynamic therapy, have been investigated in this regard (Li et al., 2023; Philip et al., 2018).

One category of natural products that has become a focus of interest is probiotics and prebiotics. Prebiotics are defined as substrates that are selectively utilized by host microorganisms, conferring a health benefit (Gibson et al., 2017). Arginine is a semi-essential amino acid that plays a role in protein synthesis, substance metabolism, immune regulation, anti-inflammatory and antioxidant activities, and various signaling pathways (Wei et al., 2023). Arginine, considered an oral prebiotic, is mainly derived from diet and host saliva and catabolized and metabolized by bacteria via the arginine deiminase system, producing ammonia as a major product. The protonation of ammonia to ammonium increases the pH, which creates a less cariogenic environment (Kuriki et al., 2021).

Arginine is widely reported to inhibit the growth of *S. mutans*, indicating its inhibitory role in the development of caries by affecting the microbiome of the oral microenvironment (Huang et al., 2017). At present, studies on the effect of arginine on *C. albicans* are limited and inconclusive. To the best of our knowledge, no study has investigated whether arginine inhibits the synergistic cariogenic effects of *C. albicans* and *S. mutans*.

In this study, we aimed to investigate whether arginine exerts an inhibitory effect on the growth of *S. mutans* and *C. albicans*, with the expectation of finding new preventive and therapeutic options for inhibiting their synergistic cariogenicity. We proposed the following null hypotheses: 1. Arginine does not inhibit the growth of *S. mutans* and *C. albicans* in single cultures; 2. Arginine does not inhibit the co-culture growth of *S. mutans* and *C. albicans*; and 3. Arginine does not inhibit the synergistic cariogenic effects of *S. mutans* and *C. albicans*.

## 2 Materials and methods

### 2.1 Strains and growth conditions

Standard wild-type *S. mutans* UA159 and *C. albicans* SC5314, inoculated from frozen glycerol stocks, were used in all experiments. *Streptococcus mutans* strains were cultivated in brain heart infusion (BHI) broth, and *C. albicans* strains were cultivated in yeast peptone dextrose (YPD) broth at 37°C under aerobic conditions with 5% CO_2_ overnight (without shaking). Cells were grown to the mid-exponential phase and harvested by centrifugation (5000 rpm, 5 min, 4°C). *S. mutans* and *C. albicans* were inoculated into BHI broth supplemented with a 1% (w/v) sucrose (BHI-S), with or without 100 mM L-arginine at the concentration for which the optical density of the suspensions at 600 nm (OD_600nm_) reached 0.005 for the subsequent experiments. BHI-S medium was autoclaved and the required amount of L-arginine (0.1742 g per 10 mL) was added aseptically to achieve a final concentration of 100 mM. The solution was then vortexed until fully dissolved, after which it was sterilized by passing it through a 0.22 µm membrane filter. The experiments were performed in triplicate.

### 2.2 Effects of arginine on planktonic cultures of *S. mutans* and *C. albicans*

#### 2.2.1 Growth curves

Planktonic growth of *S. mutans* and *C. albicans* co-cultures and monocultures, with or without arginine, was monitored at 4, 6, 8, 10, 12, 14, 16, 20, 22, and 24 h based on the optical density of the culture supernatant. By measuring the optical density at 600 nm (OD_600nm_) using a diluphotometer (Implen, Munich, Germany), microbial growth analysis was performed. The microbial culture was shaken well and added to the cuvette in a volume of 2 mL, whereas a cuvette containing 2 mL of BHI-S was used as a blank control. For microbial cultures at later stages of growth, we measured absorbance by adding a volume of 200 μL culture to 1800 μL phosphate-buffered saline (PBS) and diluting it 10-fold. The control consisted of an equal volume of BHI-S broth as the measured sample, and the total volume was brought to 2 ml with PBS. Growth curves were then plotted to assess the effect of arginine on growth.

#### 2.2.2 pH test

Changes in the pH of the supernatant during incubation can reflect the degree of acid production by both microorganisms and the effect of arginine. A pH meter (Orion 868, Thermo Fisher Scientific, Waltham, MA, USA) was used to measure the pH of the supernatants obtained from *S. mutans*, *C. albicans*, and dual-species cultures in the planktonic state after incubation for 4, 6, 8, 12, 20, and 24 h.

### 2.3 Effect of arginine on biofilm growth and cariogenicity of *S. mutans* and *C. albicans*

Biofilm growth was monitored based on the analyses of the absorbance of the cultures, crystal violet (CV) staining, colony-forming units (CFUs), and lactic acid production. Equal volumes of the suspensions of each strain were inoculated into the wells of 24-well microtiter plates (1 mL each) and 96-well microtiter plates (100 μL each). The plates were incubated at 37°C in the presence of 5% CO_2_.

#### 2.3.1 Growth curve

Biofilm growth curves were measured using 96-well plates. Growth curves were constructed by measuring OD_600nm_ using a multimode microplate reader (SpectraMax iD3, Molecular Devices, San Jose, CA, USA). The kinetic data were acquired by programming the microplate reader to automatically record the optical density at one-hour intervals. These values were then plotted as a line graph.

#### 2.3.2 Crystal violet staining

Biofilm biomass, formed in the presence or absence of arginine, was evaluated by CV staining after 4, 8, or 24 h of incubation. The biofilms were washed twice with PBS and stained with 0.1% (w/v) CV for 15 min. Thereafter, plates were washed twice with PBS to remove the unbound stain and dissolved in 600 μL of 100% ethanol for 30 min. The biofilm concentration was determined by measuring the optical density of the samples at 570 nm (OD_570nm_) (Liu et al., 2022).

#### 2.3.3 CFU measurement

After incubation for 4, 8, and 24 h, the biofilms of *S. mutans* and *C. albicans* in single- and dual-species cultures were analyzed to determine the CFU/mL of each strain. At each time point, biofilms were washed twice with PBS, were disrupted by sonication and then harvested, serially diluted, and plated on YPD agar supplemented with kanamycin and streptomycin (to culture *C. albicans* while inhibiting the growth of *S. mutans*) and BHI agar supplemented with amphotericin B (to culture *S. mutans* while inhibiting the growth of *C. albicans*). Plates were incubated at 37°C with 5% CO_2_ for 24 h, after which CFUs were counted.

#### 2.3.4 Lactic acid production assay

Mature biofilms were cultured in buffered peptone water supplemented with 0.2% (w/v) sucrose at 37°C with 5% CO_2_ for 3 h after rinsing with PBS. The concentrations of lactic acid produced by the biofilms were measured using an L-Lactic Acid Assay Kit (Nanjing Jiancheng, Nanjing, China) according to the manufacturer’s instructions (Lu et al., 2022).

#### 2.3.5 Exopolysaccharide production assay

Congo red (CR) staining was performed to evaluate the production of exopolysaccharides (EPS). After 24 h of incubation in 96-well plates, the biofilms were washed using PBS, and each well was stained with a mixture of 100 μL BHI-S and 50 μL Congo red (0.5 mM). After staining, the supernatants from each well were transferred to microtiter plates; the absorbance of the supernatants was measured at 490 nm in triplicate. Optical density values were used to measure EPS production as per the following formula: OD of EPS production = OD of blank control group supernatant CR− OD of experiment group supernatant (Kulshrestha et al., 2016).

#### 2.3.6 Fluorescence *in situ* hybridization

*S. mutans* and *C. albicans* were inoculated into confocal Petri dishes containing BHI-S with or without 100 mM arginine at an initial concentration of OD_600nm_ = 0.005, and incubated for 4 h. Thereafter, samples were fixed with 4% paraformaldehyde, eluted twice using PBS solution, and dried at room temperature.

The solutions were prepared as required for the experiment. The hybridization solution comprised 20 mM trimethylolamine hydrochloride, 0.9 M NaCl, 0.01% sodium dodecyl sulfate, and 20% deionized formamide; the pH was 7.5 and the solution was stored at 4°C. The elution solution comprised 215 mM NaCl, 20 mM tris(hydroxymethyl)aminomethane, and 5 mM ethylenediaminetetraacetic acid; the pH was 7.5 and the solution was stored at room temperature. In addition, we prepared 20x SSC solution (3 M NaCl, 0.3 M sodium citrate), which was stored at room temperature.

The *S. mutans* fluorescent probe (ATTO488-5′-ACTCCAGACTTTCCTGAC-3′, Ex 502 nm/Em 524 nm) and *C. albicans* fluorescent probe (TexasRed X-5′- GCCAAGGCTTATACTCGCT-3′, Ex 596 nm/Em 615 nm) were diluted to 100 pmol/μL with enzyme-free sterile water and added to the prepared 100 μL hybridization solution so that the final concentration of each probe was 1 pmol/μL. After vortexing and oscillating for mixing, the hybridization solution containing fluorescent probes was evenly covered with the plaque and hybridized at 46°C for 3 h. The hybridized biofilm was placed in the elution solution and eluted at 48°C for 15 min. The biofilm was eluted at 37°C for 10 min using 0.1x SSC solution, and the process was repeated twice. After drying away from light, the plaques were sealed with an anti-fade sealer and stored in a dark box for subsequent imaging analysis.

Imaging analysis was performed using a Zeiss LSM 780 laser confocal scanning microscope, with a laser emission wavelength of 488 nm/561 nm, laser power of 2.0%, and an xy-axis image size of 1024 × 1024 pixels. The whole layer of the biofilm was captured along the Z-axis of the biofilm during the imaging process, with Z-axis layers swept at an interval of 1 μm. Three images were taken from each group of samples. The Imaris 10.2 (Bitplane, Switzerland) software was used to construct the 3D structure of the biofilm and perform post-image processing. The heights of the biofilm xz and yz planes were measured to compare the thickness of the biofilms in the experimental and control groups, and to compare the differences in interspecies distances between *S. mutans* and *C. albicans*, in order to assess the effect of arginine on the interspecies adhesion of *S. mutans* and *C. albicans*.

### 2.4 Short-term treatment of arginine on biofilm growth and cariogenicity of *S. mutans* and *C. albicans*

After 4 h of incubation without arginine, the medium was changed to BHI-S medium containing 100 mM arginine, followed by incubation for 10 min. After the treatment, the medium was again changed to the original one, and the incubation was continued. This was performed to evaluate the short-term effect of arginine after 24 h.

As described in sections 2.2 and 2.3, the change in the absorbance of the solutions was measured to evaluate the planktonic growth; we also performed CV staining and measurement of the CFU counts for all the biofilms.

### 2.5 Statistical analyses

Biofilm CV staining, biomass, thickness of the biofilm, interspecies distances, EPS production, and lactic acid production were analyzed using IBM SPSS 27.0 (IBM, Armonk, NY, USA). The Shapiro–Wilk test was used to determine whether the data were normally distributed. T-tests and one-way analysis of variance (ANOVA) were used for data conforming to the normal distribution, whereas Mann–Whitney U tests and Kruskal–Wallis tests were used otherwise. Statistical significance was set at values of *p* < 0.05.

## 3 Results

### 3.1 Arginine inhibited the planktonic growth of both *S. mutans* and *C. albicans*

**Figure 1:**
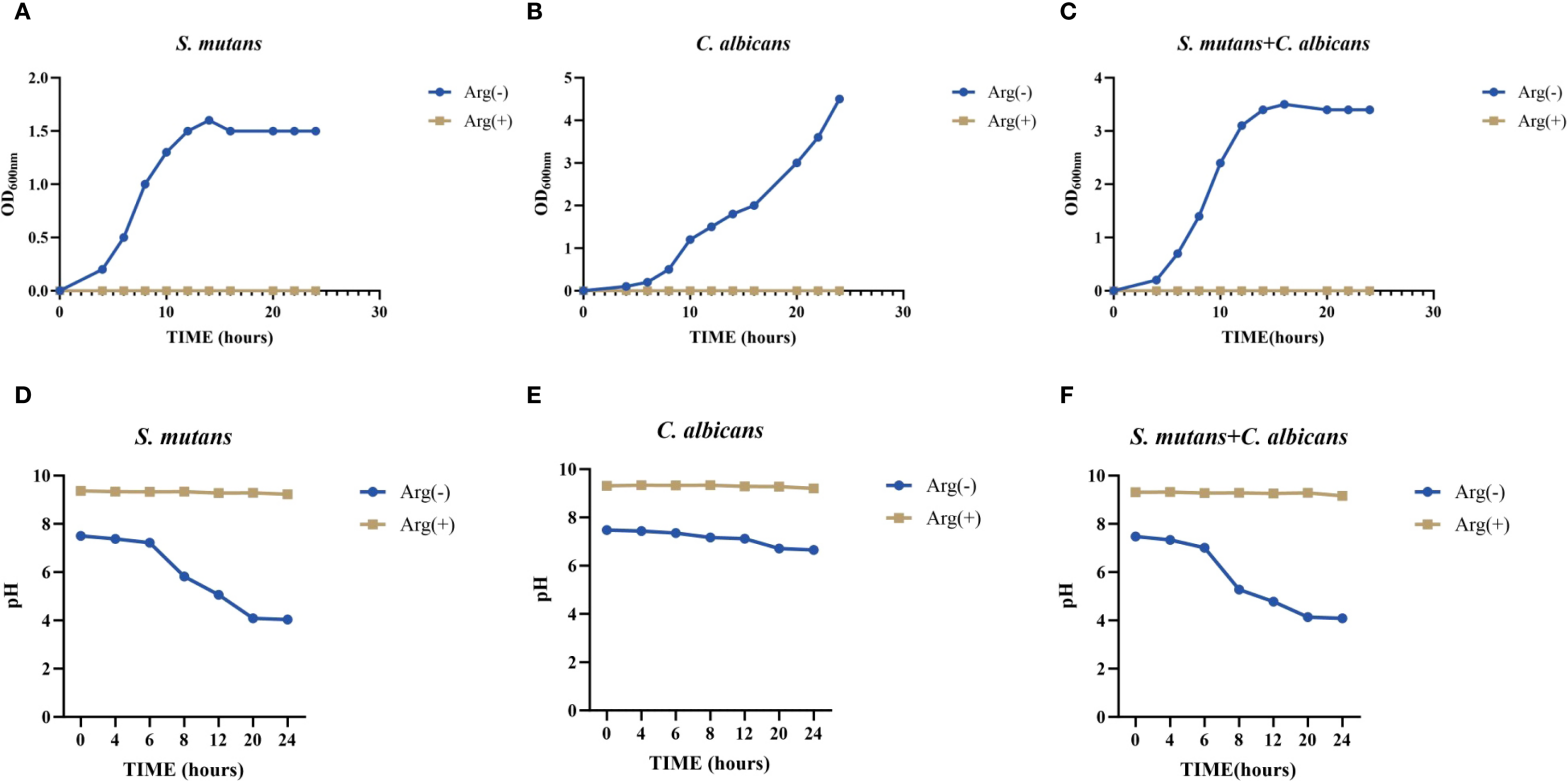
Planktonic growth curve and pH of *Streptococcus mutans* and *Candida albicans* in non-treated control group (0 mM arginine) and treated group (100 mM arginine). Planktonic growth curves of *S. mutans* **(A)**, *C. albicans* **(B)**, and dual-species **(C)** after incubation for 4, 6, 8, 10, 12, 14, 16, 20, 22, and 24 h Planktonic pH of *S. mutans* **(D)**, *C. albicans* **(E)**, and dual-species **(F)** after incubation for 4, 6, 8, 12, 20, and 24 h.

Our results showed that *C. albicans* promoted the growth of *S. mutans* when co-cultured in the planktonic state, with a decrease in the pH of the supernatant. Absorbance value measurements of the bacterial broths after 24 h incubation showed that the addition of 100 mM arginine significantly inhibited the growth of both *S. mutans* and *C. albicans*, as well as the symbiotic growth of the dual-species culture in the planktonic state (**Figures 1A–C**).

The results of pH testing of the planktonic culture solution showed that *S. mutans* had a stronger acid-producing ability than did *C. albicans*, which may lower the pH below the demineralization threshold, and could lead to the emergence of enamel demineralization, corroborating the pathogenicity of *S. mutans* and the synergistic effect of *S. mutans* and *C. albicans* in caries. In the supernatants of *S. mutans* and *C. albicans* monocultures and their co-cultures (all in the planktonic state), the addition of arginine minimized the pH decrease and maintained the pH at a level above the demineralization threshold (**Figures 1D–F**).

This suggests that arginine may reduce the effect on enamel demineralization by reducing the acid-producing properties of *S. mutans* and *C. albicans*, and by maintaining a non-cariogenic alkaline environment.

### 3.2 Arginine inhibited the formation of both *S. mutans* and *C. albicans* biofilms

**Figure 2:**
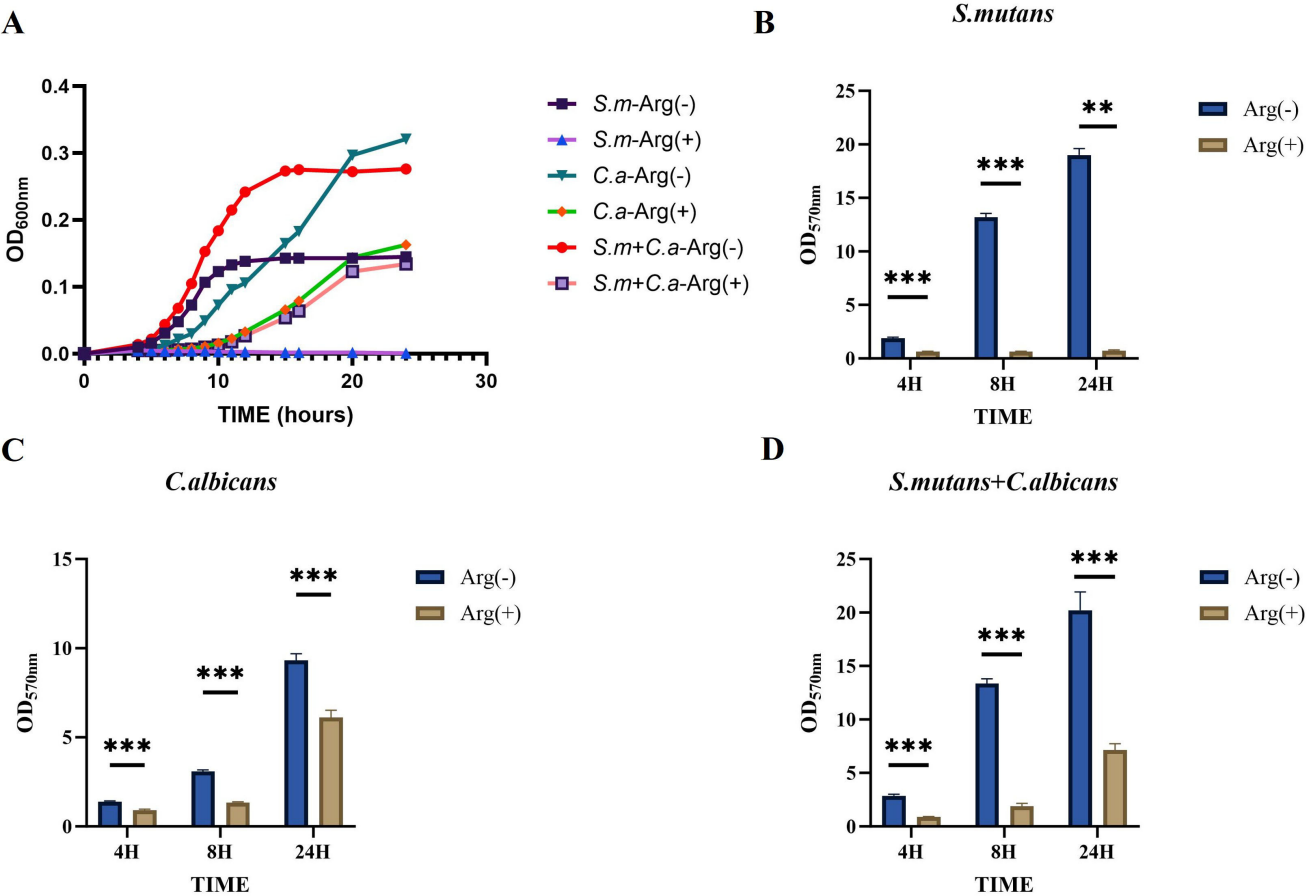
Biofilm growth of *Streptococcus mutans* and *Candida albicans* in non-treated control group (0 mM arginine) and treated group (100 mM arginine). Biofilm growth curves of *S. mutans*, *C. albicans*, and dual-species **(A)**. Biofilm mass determined by crystal violet staining of *S. mutans* **(B)**, *C. albicans* **(C)**, and dual-species **(D)**. Values are expressed as the mean and SD. Student’s *t*-test and Mann–Whitney U tests were used to compare biomass between the control and experimental groups (*p < 0.05, **p < 0.01, and ***p < 0.001).

Our results demonstrated a symbiotic promotion of growth for *S. mutans* and *C. albicans* when co-cultured in the biofilm state. The presence of *C. albicans* enabled the growth of *S. mutans*, and the co-culture biofilms exhibited thicker and larger biomass. The biofilm growth curves of *S. mutans*, *C. albicans*, and dual-species cultures showed decreased biofilm formation rates in the presence of arginine in the growth medium, with growth curves ultimately showing a reduction in the biomass, a delay in the onset of the rapid growth phase, and a slowing of the growth rate. The inhibitory effect of arginine on the growth of *S. mutans* monoculture biofilms was the most pronounced, with essentially no growth (**Figure 2A**).

The CV staining and CFU counts of single-species and double-species biofilms were performed at three selected time points, namely the early 4 h incubation, the middle 8 h incubation, and the late stage 24 h incubation stages of growth. Moreover, CV staining showed that the addition of 100 mM arginine inhibited biofilm formation (**Figures 2B–D**).

**Figure 3:**
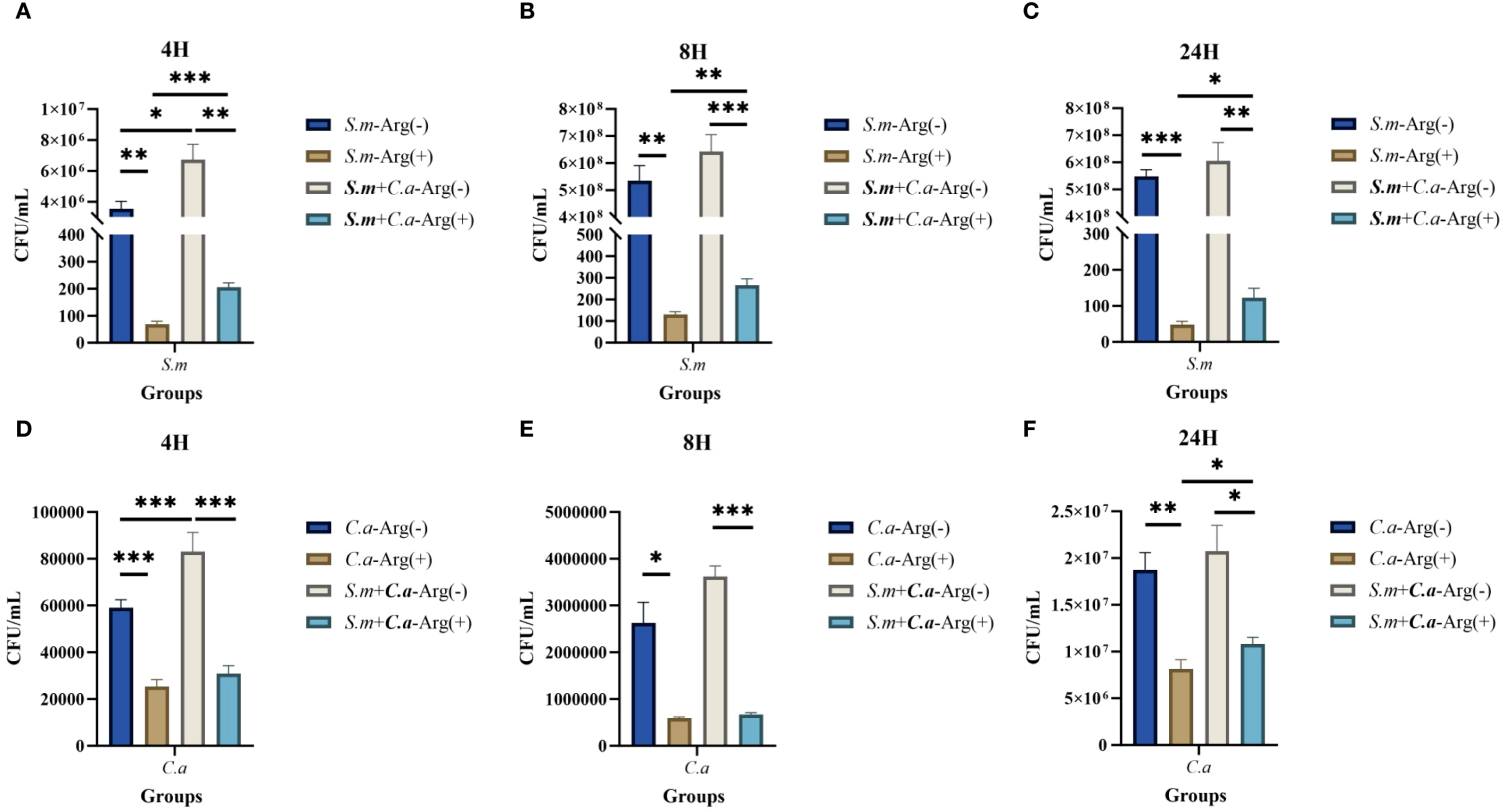
Colony forming units (CFUs) of single- and dual-species biofilms. Mean and SD of CFU/mL of *Streptococcus mutans* after incubation for 4 **(A)**, 8 **(B)**, and 24 **(C)** h and *Candida albicans* after incubation for 4 **(D)**, 8 **(E)**, and 24 **(F)** h in single- and dual-species biofilm viable cells obtained for the control group (0 mM arginine) and treated group (100 mM arginine). One-way ANOVA and Kruskal–Wallis tests were used to compare the control and experimental groups (*p < 0.05, **p < 0.01, and ***p < 0.001).

The CFU counts of *S. mutans*, *C. albicans*, and dual-species biofilms observed after incubation for 4, 8, and 24 h were used to determine the effects of arginine on biofilm biomass. Our results showed that co-culture of *S. mutans* and *C. albicans* resulted in more colonies of *S. mutans* and *C. albicans* compared with the respective number of colonies in monoculture biofilms at the three assayed time points. Arginine reduced the counts of *S. mutans* and *C. albicans* in the biofilms. The counts of both *S. mutans* and *C. albicans* in the single-species biofilm were significantly lower than those of the two organisms in the dual-species co-culture biofilm (**Figure 3**). CFU counts of *S. mutans* and *C. albicans* were consistent with the previous results of biofilm CV staining, both of which demonstrated that the addition of arginine reduced the biofilm biomass of microorganisms, resulting in a reduction in biofilm production. Our results indicate that arginine may inhibit the formation of cariogenic biofilm by decreasing the biomass of biofilm, thereby achieving a caries-inhibiting effect.

**Figure 4:**
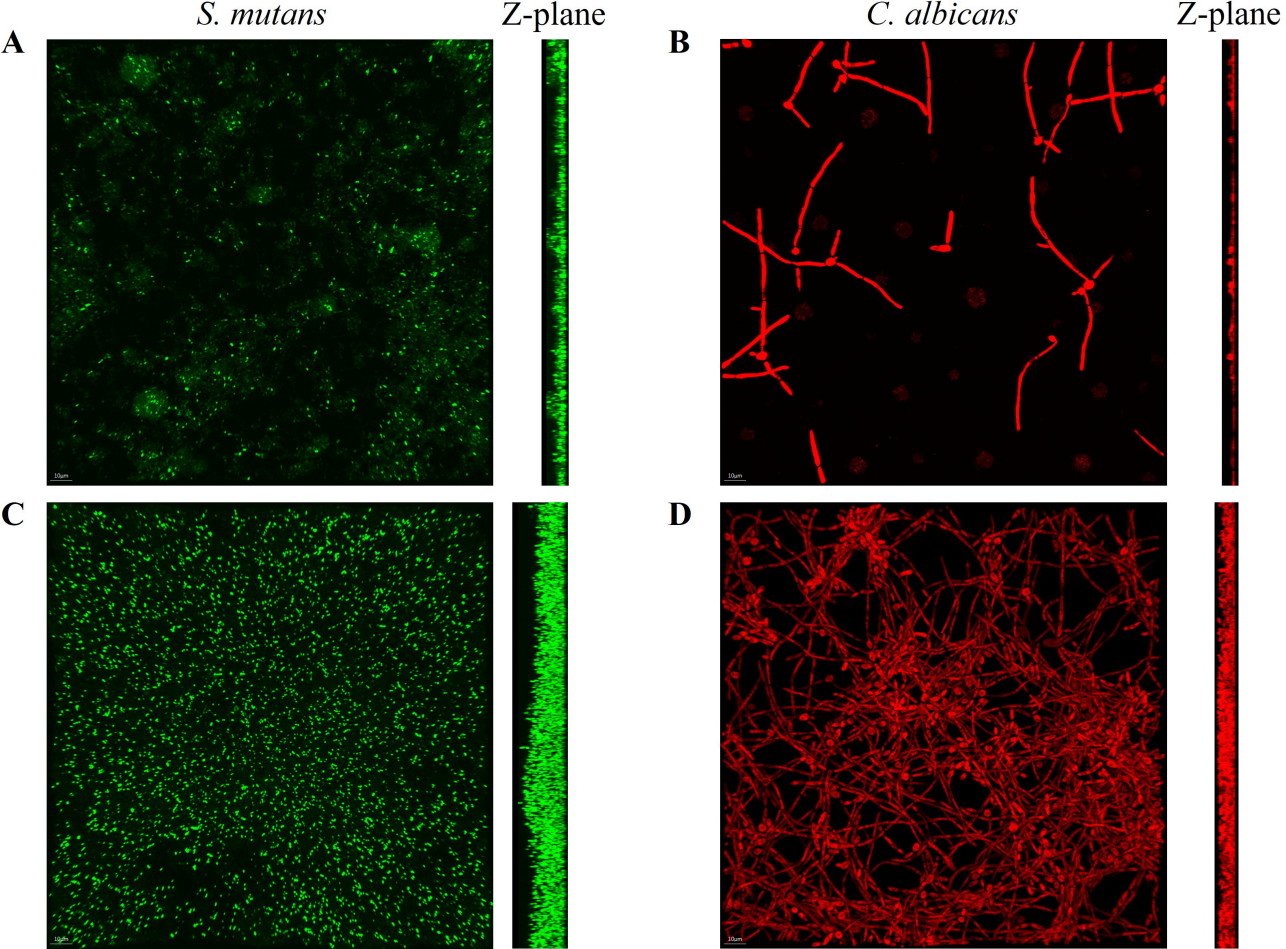
Fluorescence *in situ* hybridization staining images of biofilms of *Streptococcus mutans* biofilms in the experimental group **(A)** and control group **(C)**, and *Candida albicans* biofilms in the experimental group **(B)** and control group **(D)** after 4 h of incubation, with the Z-plane denoting biofilm thickness.

**Figure 5:**
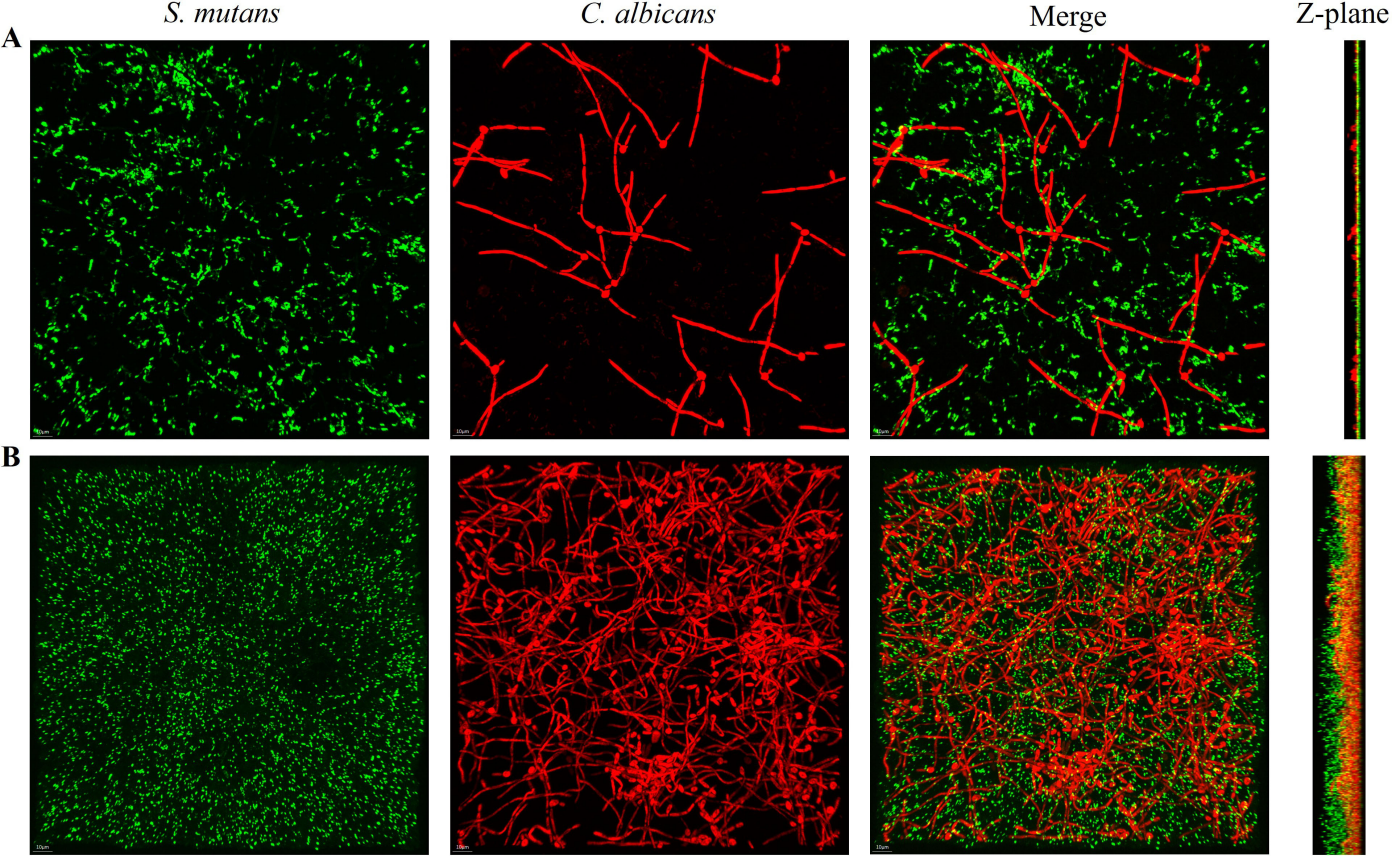
Fluorescence *in situ* hybridization staining images of *Streptococcus mutans* and *Candida albicans* co-cultured biofilms with the addition of 100 mM arginine **(A)** and in the control group **(B)** after 4 h of incubation, with the Z-plane indicating biofilm thickness.

**Figure 6:**
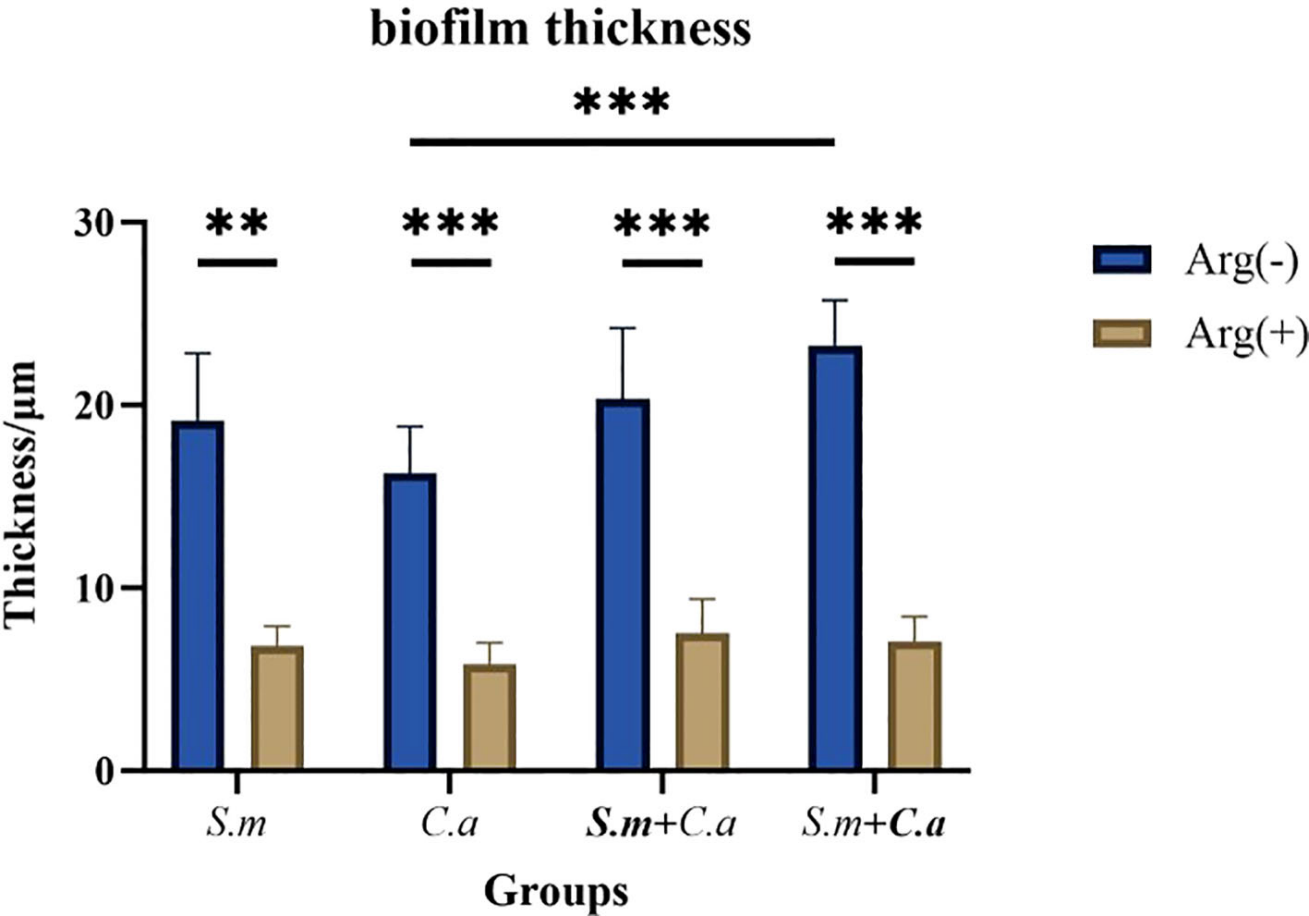
Thickness measurements of *Streptococcus mutans* and *Candida albicans* monoculture and co-culture biofilms after 4 h of incubation with 100 mM and 0 mM arginine (*p < 0.05, **p < 0.01, and ***p < 0.001).

**Figure 7:**
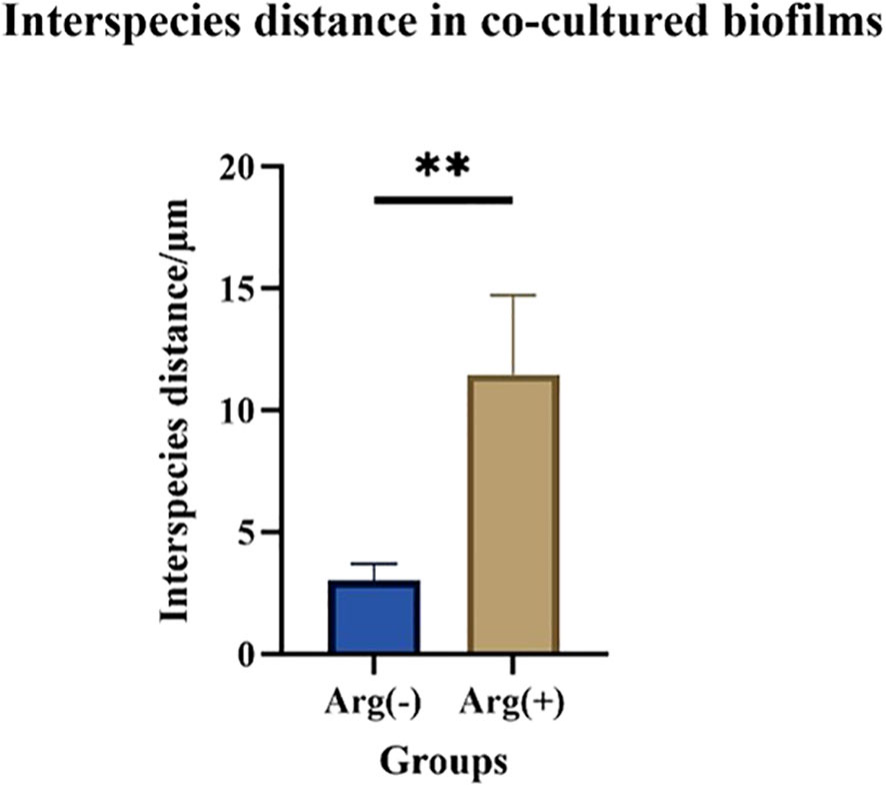
Interspecies distance measurements of *Streptococcus mutans* and *Candida albicans* in co-cultured biofilms after 4 h of incubation with 100 mM and 0 mM arginine (**p < 0.01).

FISH staining was performed on single- and dual-species biofilms of *S. mutans* and *C. albicans* after 4 h of incubation. Biofilms were observed using a confocal fluorescence microscope. Both monoculture (**Figure 4**) and dual-culture (**Figure 5**) biofilms of *S. mutans* and *C. albicans* showed a decrease in biomass after the addition of 100 mM arginine, and this result was in line with the trend of the results of biofilm CFU counting. Moreover, biofilm thicknesses were significantly decreased after the addition of 100 mM arginine. The reduction of both biomass and biofilm thickness indicated that arginine could inhibit biofilm formation (**Figure 6**). Simultaneously, the interspecies distance of *S. mutans* and *C. albicans* in the co-cultured biofilm increased after the addition of 100 mM arginine (**Figure 7**).

The biomass of *S. mutans* and *C. albicans* in the biofilm was reduced, the physical contact between the strains was sparse, and the biofilm became loosely packed. The synergistic cariogenic effect between *S. mutans* and *C. albicans* included their physical adhesion, and their close association contributed to the biomass accumulation of the co-cultured biofilm and stabilization of the biofilm structure, which may also be one of the ways that arginine inhibited the co-cultivated biofilm of *S. mutans* and *C. albicans*.

**Figure 8:**
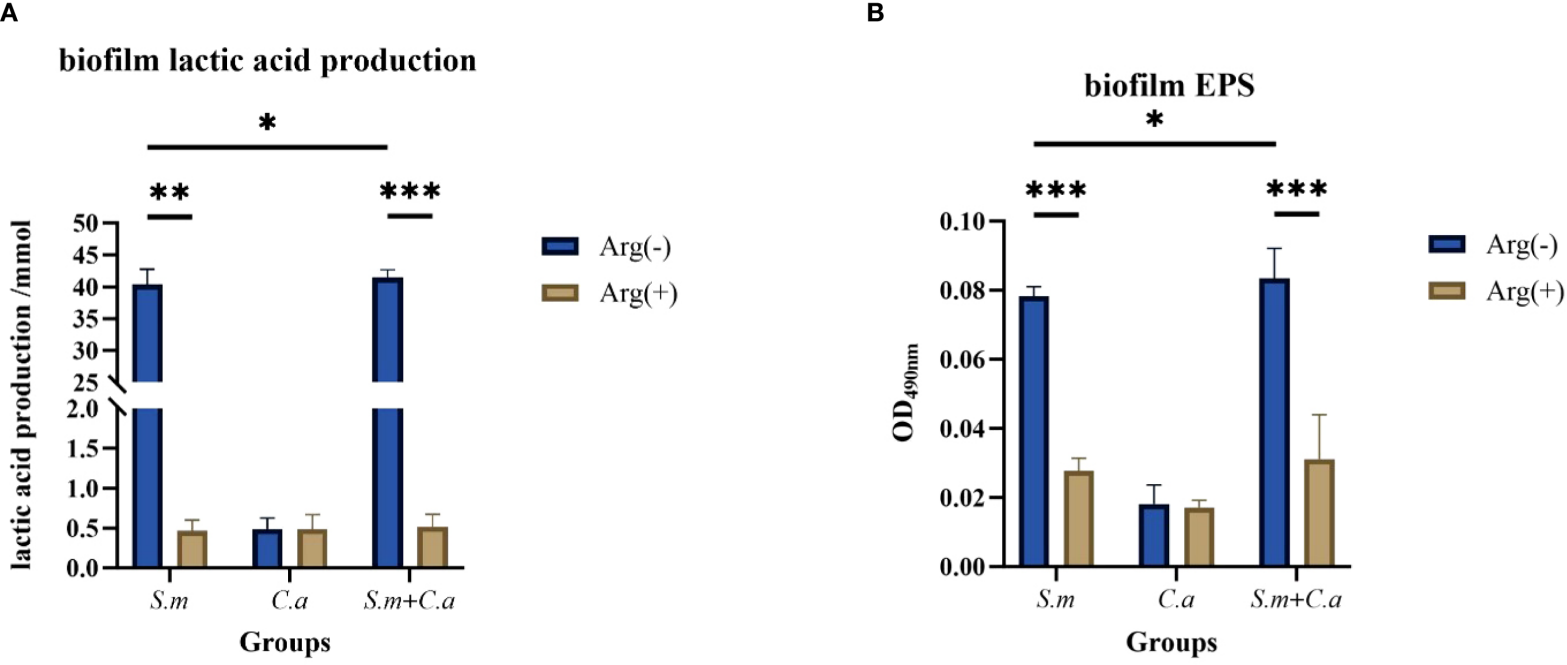
Lactic acid **(A)** and extracellular polysaccharide **(B)** production in single- and dual-species biofilms in the control group (0 mM arginine) and experimental group (100 mM arginine). Values are expressed as the mean and SD. Student’s *t*-test and Mann–Whitney U tests were used to compare biomass between the control and experimental groups (*p < 0.05, **p < 0.01, and ***p < 0.001).

The metabolism of *S. mutans* for acid production from dietary sugars and the production of extracellular polysaccharides (EPSs) for biofilm generation are crucial indicators of its cariogenicity. The production of lactic acid and EPSs by co-cultured biofilm was increased compared with that observed in the *S. mutans* single-species biofilm, thus increasing in the cariogenicity of the co-cultured biofilm, in line with the synergistic cariogenic effects of *S. mutans* and *C. albicans* as reported in previous studies. *S. mutans* and dual-species biofilms exhibited higher lactic acid production than *C. albicans* biofilms. L-Arginine (100 mM) inhibited biofilm lactate production, substantially reducing levels compared with those observed without arginine (**Figure 8A**). The addition of arginine decreased the level of EPSs produced, leading to the suppression of biofilm formation. This finding was consistent with the biofilm CV staining results, suggesting that arginine had an inhibitory effect on the cariogenicity of the co-cultured biofilms (**Figure 8B**).

In summary, the addition of arginine to the culture medium inhibited the formation of *S. mutans* and *C. albicans* single- and dual-species biofilms, resulting in the suppression of biofilm formation and a decrease in biofilm biomass, as well as the production of extracellular polysaccharides and lactic acid within the biofilms.

### 3.3 Short-term arginine treatment inhibited the growth of both *S. mutans* and *C. albicans*

**Figure 9:**
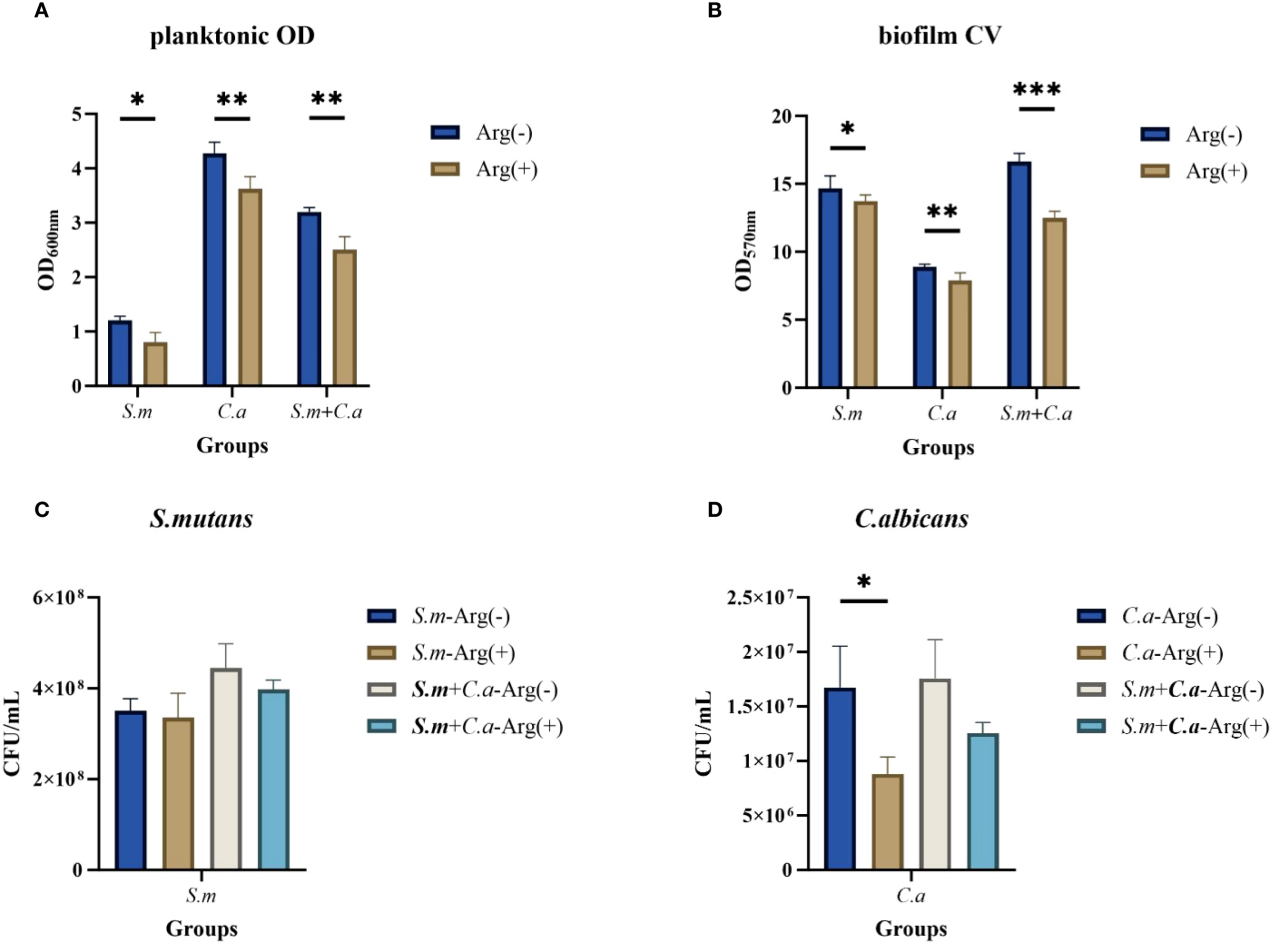
Short-term effect of arginine on planktonic and biofilm growth of *Streptococcus mutans* and *Candida albicans*. Planktonic solution absorbance in non-treated control group (0 mM arginine) and treated group (100 mM arginine, treated for 10 min after 4 h incubation) **(A)**. Biofilm mass determined by crystal violet staining **(B)** and CFU/mL of *S. mutans* **(C)** and *C. albicans* **(D)** in single- and dual-species biofilm viable cells obtained for the control group (0 mM arginine) and experimental group (100 mM arginine, treated for 10 min after 4-h incubation). Values are expressed as the mean and SD. Student’s *t*-test and Mann–Whitney U tests were used to compare biomass between the control and experimental groups (*p < 0.05, **p < 0.01, and ***p < 0.001).

We treated *S. mutans* and *C. albicans* with 100 mM arginine for 10 min after 4 h of incubation. Short-term treatment with arginine similarly inhibited the planktonic and biofilm growth of both single- and dual-species cultures but was not as effective as the long-term treatment of adding arginine to the culture medium. These results suggest that arginine inhibits the planktonic growth of *S. mutans*, *C. albicans*, and the dual-species culture (**Figure 9A**). Biofilm CV staining and analysis of the CFU counts (**Figures 9B–D**) demonstrated that short-term treatment of biofilms with arginine also reduced biofilm formation, resulting in a decrease in biofilm biomass. However, some of these results demonstrated no statistically significant differences.

## 4 Discussion

In the present study, we demonstrated the potential of arginine as a prebiotic agent that may serve as a novel caries control measure by exerting inhibitory effects on cavity-causing microorganisms. Our results showed that the use of arginine as a prebiotic inhibited both mono- and dual-species growth of *S. mutans* and *C. albicans* and reduced the production of EPSs and lactic acid in biofilms. Thus, our null hypothesis that arginine does not affect the growth and synergistic cariogenic effects of *S. mutans* and *C. albicans* is rejected.

*S. mutans* and *C. albicans* have been shown to co-exist in several models of oral diseases, especially caries, and are important in disease development. Although fluoride is a known standard of care for the prevention of caries, its effects on oral biofilms are limited (Dang et al., 2016). The use of broad-spectrum antimicrobials, such as chlorhexidine, may alter the composition of the oral flora by non-selectively destroying bacteria and allowing antimicrobial-resistant bacteria to flourish. Given the synergistic cariogenic effects between *S. mutans* and *C. albicans*, approaches targeting microorganisms or cross-kingdom interactions may be effective in treating or preventing caries.

Therapeutic approaches targeting *S. mutans* and *C. albicans* biofilms include natural compounds, antimicrobial peptides, nanomaterials, antimicrobial photodynamic therapy, and combination therapy, all of which have demonstrated good inhibitory effects as antimicrobial therapies (Khan et al., 2021; Li et al., 2023). Arginine has shown promise in preventing and treating dental caries in several studies. Arginine is metabolized by five main pathways: (1) to creatine and homoarginine by arginine glycine amidinotransferase; (2) biosynthesized to guanidine butyramine and carbon dioxide by decarboxylation of arginine decarboxylase; (3) produced to citrulline and nitric oxide by nitric oxide synthase; (4) decomposed to ornithine and urea by arginases; and (5) metabolized to ammonia and citrulline by arginine deiminase (Wei et al., 2023). Arginine can be metabolized by microorganisms with arginine deiminase systems, such as *Streptococcus gordonii*, to produce ammonia, which increases the environmental pH and reduces the occurrence of enamel demineralization (Jing et al., 2022). In contrast, *S. mutans* does not have an arginine deiminase system and cannot metabolize arginine. Most of these studies focused on the effects of arginine against *S. mutans* showed that arginine negatively impacts *S. mutans* biofilm formation ability, pathogenicity, metabolism, and tolerance to environmental stressors (Huang et al., 2017). Several studies have examined the effects of the addition of arginine to fluoridated toothpaste and found that a fluoridated toothpaste containing 1.5% arginine exhibited more prominent anti-caries effects (Yin et al., 2013; Li et al., 2015). Arginine-containing toothpaste favorably modifies the bacterial composition to a healthier community (Nascimento et al., 2014). In addition, arginine has been added to products such as mouth rinses (Wang et al., 2012; Yu et al., 2017), varnishes (Shapira et al., 1994), and confections (Acevedo et al., 2008) to study its caries-controlling effects. Furthermore, 8% arginine has been reported to be effective in treating dentin hypersensitivity (Hirsiger et al., 2019).

*C. albicans* can encode arginases that metabolize arginine (Schaefer et al., 2020). However, relatively few studies have focused on the effects of arginine on the growth of *C. albicans* and have indicated different effects than those on *S. mutans*. Ghosh et al. demonstrated that arginine supplementation stimulates hyphal growth in *C. albicans* (transition from yeast to hyphal form is a critical virulence factor in *C. albicans*) (Ghosh et al., 2009). Another study reported that the addition of 0.2% arginine promoted cross-kingdom interactions between *C. albicans* and *Actinomyces viscosus* in root caries (Xiong et al., 2022). In contrast, Koopman et al. demonstrated that arginine supplementation enhanced the resilience of the oral microenvironment against acidification and suppressed *C. albicans* outgrowth (Koopman et al., 2015). However, to the best of our knowledge, no prior studies have examined the effects of arginine on the combined growth of *S. mutans* and *C. albicans*.

Our study focused on whether arginine has an inhibitory effect on *S. mutans* and *C. albicans*, with the goal of guiding the clinical application of arginine and exploring novel methods for inhibiting the development of caries. Previous studies reported that toothpastes supplemented with 1.5% arginine were used for the prevention of dental caries. Similarly, we used 100 mM arginine and investigated its effects on the growth of *S. mutans*, *C. albicans*, and their dual-species co-cultures. In the present study, a high concentration of arginine (100 mM) inhibited the growth of both single- and dual-species cultures in the planktonic and biofilm states. Biofilm CV staining, CFU quantification, and FISH showed that arginine inhibited biofilm formation by reducing both biomass and physical adhesion. These results lead to the rejection of our null hypotheses, indicating that the effect of arginine not only inhibits the growth of *S. mutans* and *C. albicans* in both single- and dual-species cultures but also suppresses the synergistic cariogenic effects of these organisms. The effect of arginine on *S. mutans* was consistent with that reported in previous studies. Our results also demonstrated that arginine inhibited the growth of *C. albicans*, consistent with prior findings. However, discrepancies with certain previous findings suggest that the effect of arginine on *C. albicans* growth is concentration-dependent; higher concentrations (such as the 100 mM arginine used in this study) appear to exert stronger inhibitory effect.

Our results showed that *S. mutans* and *C. albicans* co-culture enhanced the growth and cariogenic properties of *S. mutans*. Previous studies have demonstrated that the presence of *C. albicans* increases *S. mutans* metabolism, including the upregulation of genes involved in glycolytic carbohydrate metabolism (*eno*) and galactose metabolism (*lacC* and *lacG*), as well as genes related to acid production (*ldh*) and acid stress tolerance (*fabM* and *atpD*). Additionally, the downregulation of genes associated with ammonia production (*arcA* and *ureC*) allows the pH to drop below the demineralization threshold (Du et al., 2021; Xiao et al., 2023). *S. mutans* is a major producer of extracellular polysaccharides in dental plaque biofilms, converting dietary sucrose to extracellular glucans primarily through glucosyltransferases (Gtfs) (Krzyściak et al., 2014). Gtfb secreted by *S. mutans* binds tightly to the mannan layer of *C. albicans*, resulting in the production of a large amount of extracellular α-glucan on the fungal surface, contributing to the bacterial-fungal association and biofilm formation (Hwang et al., 2017; Bowen et al., 2018; Falsetta et al., 2014).

Additionally, arginine has been shown to increase the pH of the oral microenvironment, as its metabolism leads to the production of ammonia, and downregulates genes associated with the production of extracellular polysaccharides (*gtfB*) in *S. mutans*, thereby affecting its cariogenic properties (Chakraborty and Burne, 2017). Our results indicate that the production of extracellular polysaccharides and lactic acid within the dual-species biofilm decreased after the addition of arginine, while also maintaining a higher pH, which reduces the initiation and progression of demineralization and caries. These findings suggest that arginine has an inhibitory effect on the co-cultured biofilm and reduces the pathogenicity of *S. mutans* and *C. albicans*, representing a novel approach to preventing caries.

Our results showed that the co-culture of *S. mutans* and *C. albicans* enhanced the growth of both organisms and the cariogenic properties of the co-cultured biofilms. The inhibition of growth of both *S. mutans* and *C. albicans*, along with the reduction of biomass, physical adhesion, EPS production, and lactic acid production in the co-cultured biofilms after the addition of arginine, may explain how arginine reduces the cariogenicity of *S. mutans* and *C. albicans*, presenting a new strategy for caries prevention.

Arginine exerts different inhibitory effects on *C. albicans* and *S. mutans*, presumably due to their different modes of action toward fungi and bacteria. In *S. mutans*, arginine significantly impacts physiological homeostasis and gene regulation. Arginine inhibits growth, suppresses virulence and compromises stress tolerance (Chakraborty and Burne, 2017). Recent evidence shows that arginine can weaken the *S. mutans* cell wall, ultimately causing lysis (Liu et al., 2022).

In contrast, the effect of arginine against *C. albicans* is believed to resemble the action of cationic surfactants, primarily targeting the plasma-membrane lipids (Fait et al., 2023). As arginine has a more profound and multifaceted impact on the physiology and transcriptome of *S. mutans*, its inhibitory effect is more pronounced. Consequently, when *S. mutans* and *C. albicans* are co-cultured, the pronounced suppression of the former disrupts their cross-kingdom synergy and markedly restricts the overall growth of the dual-species community.

We also investigated whether the short-term application of arginine affected the growth of *S. mutans* and *C. albicans*; the inhibitory effects of arginine against the growth of these bacteria were similar. Short-term treatments reduced the biomass of *S. mutans* and *C. albicans* in the planktonic state. Similarly, in the biofilm state, a reduction in biofilm biomass was noted, although the CFU counts of *S. mutans* and *C. albicans* showed no statistically significant differences. It is encouraging to note that short-term treatment with arginine also inhibited the formation of *S. mutans* and *C. albicans* biofilms. This suggests adding arginine to toothpaste, mouthwash, and other oral cleansing products, in conjunction with regular oral hygiene, could be effective in inhibiting the growth of caries-causing bacteria and preventing the development of caries. Further research is needed to determine the duration and effectiveness of the short-term application of L-arginine.

Arginine may help prevent dental caries due to its effect on the growth of both *S. mutans* and *C. albicans*. This inhibition results in a decrease in biofilm biomass, which, in turn, reduces cariogenic biofilm production. Moreover, arginine affects the physical adhesion of these microbes, thereby affecting the formation of a symbiotic biofilm. Arginine may also suppress the expression of virulence factors in *S. mutans*, leading to reduced production of EPSs and lactic acid, as well as maintaining a higher pH. Collectively, these changes contribute to a decrease in the onset and progression of demineralization and caries. Moreover, the cross-kingdom cariogenic effect of *S. mutans* and *C. albicans* can enhance the cariogenicity of *S. mutans*. Arginine could reduce the production of extracellular polysaccharides and lactic acid, which become more abundant during co-culture, as reflected in the reduced cariogenicity of *S. mutans*. This suggests that the inhibitory effect of arginine may stem from its action on the cross-kingdom interactions between *S. mutans* and *C. albicans*. However, further studies are required to determine whether the mechanisms underlying the inhibitory effects of arginine are associated with its action on the growth of a single microorganism or its effect on the cross-kingdom interactions between both microorganisms. Moreover, future studies should investigate the mechanisms by which the inhibitory effect of arginine are mediated through the regulation of genes in these two pathogens.

## 5 Conclusion

L-Arginine inhibited the growth and biofilm formation of *S. mutans* and *C. albicans*, both monocultured and co-cultured. Moreover, L-arginine suppressed the bacterial growth-associated reduction in pH, as well as the production of EPSs and lactic acid. Our findings suggest that L-arginine can serve as a potential candidate to inhibit the synergistic cariogenicity of *S. mutans* and *C. albicans*.

## Data Availability

The raw data supporting the conclusions of this article will be made available by the authors, without undue reservation.
